# Potential efficacy of crizanlizumab in treating priapism in sickle cell disease: A case report

**DOI:** 10.1002/ccr3.8585

**Published:** 2024-05-10

**Authors:** Mohammed Abdulgayoom, Mohammad S. Afana, Awni Alshurafa, Mohamed A. Yassin

**Affiliations:** ^1^ Department of Hematology National Center for cancer care and Research, HMC Doha Qatar

**Keywords:** acute chest syndrome, crizanlizumab, infusion‐related reactions, pain crises, priapism, vaso‐occlusive crises

## Abstract

This report documents the treatment of a 41‐year‐old male with sickle cell disease (SCD) and repeated stuttering priapism using crizanlizumab, which alleviated the priapism but induced a significant vaso‐occlusive crisis during the second infusion. Encouragingly, no subsequent vaso‐occlusive crises occurred. However, the potential for infusion‐related adverse events warrants close supervision. Further research is necessary to explore its full benefits on priapism management.

## 1 INTRODUCTION

Sickle cell disease (SCD) is a group of inherited diseases characterized by the presence of hemoglobin S (Hb S), either by homozygosity for the sickle mutation (Hb SS) or by mixed heterozygosity with another beta‐globin variant (e.g., sickle beta thalassemia, Hb SC disease). It is one of the most common hemoglobinopathies, especially in Middle Eastern countries. The hallmarks of SCD are recurrent vaso‐occlusive crises and hemolytic anemia. Vaso‐occlusive crises manifest as acute pain crises, acute chest syndrome, and priapism—they are mainly caused by obstructions of the microcirculation and lead to tissue hypoxia and severe pain.^1^, ^2^, ^3^

Priapism is defined as a persistent erection of the penis that is not associated with sexual interest or desire. It can occur with low flow (ischemic and vaso‐occlusive) or high flow (non‐ischemic). In some series, it is estimated that 35%–45% of men with sickle cell disease (SCD) are affected by priapism. Ischemic episodes lasting ≥4 h (severe episodes) are of particular concern as they carry a high risk of permanent tissue damage from penile compartment syndrome. There is another variant of ischemic priapism, known as stuttering priapism, which is characterized by brief, recurrent episodes of transient, self‐limited priapism.^4^, ^5^

Recent therapeutic advances have seen the approval of crizanlizumab, a monoclonal antibody targeting P‐selectin, by the FDA in 2019 for reducing the frequency of SCD‐related vaso‐occlusive crises. Nonetheless, the efficacy of crizanlizumab in managing SCD‐associated priapism has yet to be thoroughly investigated, underscoring a vital area for further clinical exploration.^6^

## 2 CASE PRESENTATION

A 41‐year‐old man with known sickle cell anemia (Hb SS) who had a history of frequent vaso‐occlusive crises. In the year prior to starting treatment with crizanlizumab, the patient had three pain crises at home without visiting the emergency department, three pain crises for which he was admitted to hospital, one crisis of acute chest syndrome and recurrent episodes of stuttering priapism that occurred almost weekly, each episode lasting approximately 3–5 min and having a pain score of 5–6 and usually resolving spontaneously. During this time he continued to receive folic acid 5 mg daily, hydroxyurea 1000 mg daily and an increasing requirement for narcotics, so the patient was offered to increase the dose of hydroxyurea, which he refused.

## 3 TREATMENT

In view of the recurrence of severe vaso‐occlusive crises within one‐year, prophylactic treatment with crizanlizumab at a dose of 5 mg/kg was started. The first loading dose was administered without complications. During the infusion of the second loading dose, he developed severe pain with a pain score of 8 out of 10, so the dose was administered over 1 h instead of 30 min, and he required paracetamol intravenously and morphine 5 mg subcutaneously. The third dose was administered over 1 h and subsequent doses over 30 min without complications.

## 4 OUTCOME AND FOLLOW‐UP

Two years have passed since starting treatment with crizinalizumab, apart from a hospitalization for a chest infection, during which he received intravenous fluids, intravenous antibiotics, and painkillers. He did not require any further hospitalization. His need for narcotic medications decreased significantly until they were discontinued. The stuttering priapism improved significantly and disappeared completely last year.

Initially, the patient expressed reluctance at the prospect of monthly hospital visits for the infusion of crizanlizumab. However, after observing the positive outcomes of the treatment, he became fully compliant and receptive to the ongoing monthly infusions. The shift in the patient's perspective toward the treatment underscores the effectiveness of crizanlizumab in managing his condition.

## 5 DISCUSSION

Of note in this case report is the fact that crizanlizumab, originally approved by the FDA for the treatment of sickle cell‐associated vaso‐occlusive crisis based on the SUSTAIN trial,^6^ appears to have played an important role in the complete disappearance of priapism in our patient. This finding, although an isolated case, provides a new direction for the study and treatment of priapism in SCD patients and warrants further investigation using real‐world data.

The infusion‐related reaction (IRR) that occurred in our patient during the second crizanlizumab infusion, while concerning, is consistent with previous reports of rare but still significant adverse events during the initial phase of this treatment regimen. In our patient's experience, the occurrence of IRR is not indicative of a recurrence of the episode and does not compromise the long‐term benefit of treatment. This finding should encourage physicians to consider continuing crizanlizumab treatment even after a single IRR event, no doubt after careful risk–benefit assessment.^7^, ^8^ However, our case emphasizes the urgent need for guidance on the management of such infusion‐related reactions.

The overall results of crizanlizumab administration in our patient were positive, confirming its benefit in the management of vaso‐occlusive crises in SCD and its potential benefit in stuttering priapism. Nevertheless, future studies with a larger cohort and longer follow‐up periods are urgently needed to validate these results and to better understand the unseen facets of crizanlizumab therapy.

## 6 CONCLUSION

In conclusion, our case shows that a single occurrence of infusion‐related reactions with crizanlizumab does not necessarily equate to recurrence. Therefore, the long‐term overall efficacy of the drug must be considered before a decision is made to discontinue it prematurely. In the specific case of our SCD patient, treatment with crizanlizumab showed hopeful results in stuttering priapism, an observation that needs to be confirmed by other similar cases and real‐world evidence.

## AUTHOR CONTRIBUTIONS

**Mohammed Abdulgayoom:** Writing – original draft; writing – review and editing. **Mohammad S. Afana1:** Writing – original draft. **Awni Alshurafa:** Resources; writing – review and editing. **Mohamed A. Yassin:** Supervision.

## FUNDING INFORMATION

Case report publication charge will be covered by Qatar national Library.

## CONFLICT OF INTEREST STATEMENT

The authors have no conflicts of interest.

## ETHICS STATEMENT

This case was approved by the Hamad Medical Corporation's Medical Research Center.

## CONSENT

Written informed consent was obtained from the patient to publish this report in accordance with the journal's patient consent policy.

## Data Availability

Data and materials are available on reasonable request.
